# Supplementary material for: Synthesis of Lightweight Renewable Microwave-Absorbing Bio-Polyurethane/Fe3O4 Composite Foam: Structure Analysis and Absorption Mechanism
Source: Int J Mol Sci. 2022 Oct 14;23(20):12301. doi: 10.3390/ijms232012301 (PMC9603621; doi:10.3390/ijms232012301)
Supplement: Supplementary file 1 [file ijms-23-12301-s001.zip › ijms-1937769-supplementary.pdf]

Supplementary Material

1. The composition and degree of unsaturation of *Sapiumsebiferum* kernel oil(SSO) and other common vegetable oils

The chemical components of common vegetable oil fatty acids are listed in table S1.Among them, the content of unsaturated fatty acids in SSO excels 90%, and its double bonds per molecule attain 6.6, and the iodine value as high as 186.8 g/100 g, which are much higher than those of other common vegetable oils. It eliminates competition for human food and feed, that can be used as raw materials to prepare polyols (SSP) in the synthesis of new polyurethane.

Table S1 Fatty acid components and degree of unsaturationof SSO and common vegetable oils

Oils	Double bonds	Fatty acid composition (%)					Iodine value
		C16:0	C:18:0	C:18:1	C:18:2	C:18:3	(g/100g)
Castor	3.0	2.0	1.0	7.0	3.0	0.5	81−91
Corn	4.5	10.9	2.0	25.4	59.6	1.2	118−128
Linseed	6.6	6.0	4.0	22.0	16.0	52	177
Olive	2.8	9.0	2.7	80.3	6.3	0.7	76−88
Palm	1.7	44.4	4.1	39.3	10.0	0.4	50−55
Soybean	4.6	10.6	4.0	23.3	53.7	7.6	123−139
Rapeseed	3.8	3.8	1.2	18.5	14.5	11.0	100−115
Sunflower	4.7	7.0	4.5	18.7	67.5	0.8	125−140
Canola	3.9	4.0	1.8	60.9	21.0	8.8	100−115
Tung	6.5	3.4	3.7	6.3	7.5	71.1	163−173
Cottonseed	3.9	21.6	2.6	18.6	54.4	0.7	98−118
Peanut	3.4	11.1	2.4	46.7	32.0	-	84−100
**SSO**	6.6	7.1	2.0	14.2	29.8	42.7	186.8

2. The preparation progress of SSP-based BPU foam

The polyol (SSP) is generated from SSO according to a previous method. The bio-basedpolyurethane foam(BPUF) is prepared by one step foaming process, and the

reaction equation is prepared as the follow Scheme S1. The molar ratio of -OH in SSP, PEG-400 and water to -NCO in pMDI was 1:1.1. TEA with a 2.5 wt% and SnOct with a 1 wt% of SSP were catalyst and cocatalyst, respectively. Silicone oil with a 2 wt% of SSP acted as a foam stabilizer. The process can be described briefly as follows. First, the various components which include SSP, PEG-400, water, TEA, SnOct, and silicone oil were mixed at a certain ratio and then stirred evenly for 10 mins. Next, Fe_3O_4 particles with a 30 wt % within the matrix were gradually added and the mixture was stirred for another 20 mins. Finally, the proportional pMDI was rapidly poured in and vigorously stirred for 10 secs, which enabled production of a uniform mixture.

Composite coating film that contains a certain dosage of mBPUF powder is prepared according to a strategy based on microstructure design and effective compounding, the molding processes the magnetic BPU foams and its composite film is shown in the Scheme S2. A certain mole ratio of SSP and IPDI (-OH: -NCO = 1: 1.1) and 2 wt% DBTDL were transferred into a three-neck flask and then mixed with a mechanical stirrer at 50 °C for 1 h in the purge of nitrogen. The mBPUF powders with 30 wt% of SSP were dispersed into the DCM and placed into an ultrasonic bath for 1 h. Next, the suspension was added into the matrix, and it reacted for 2 h. The complex system was then vacuumed to unsought solvent for 10 mins, and it was next poured into a polytetrafluoroethylene (PTFE) mold for solidification at 60 °C for 8 h. The pure BPU was synthesized using the same method, but without adding the magnetic foam powders.

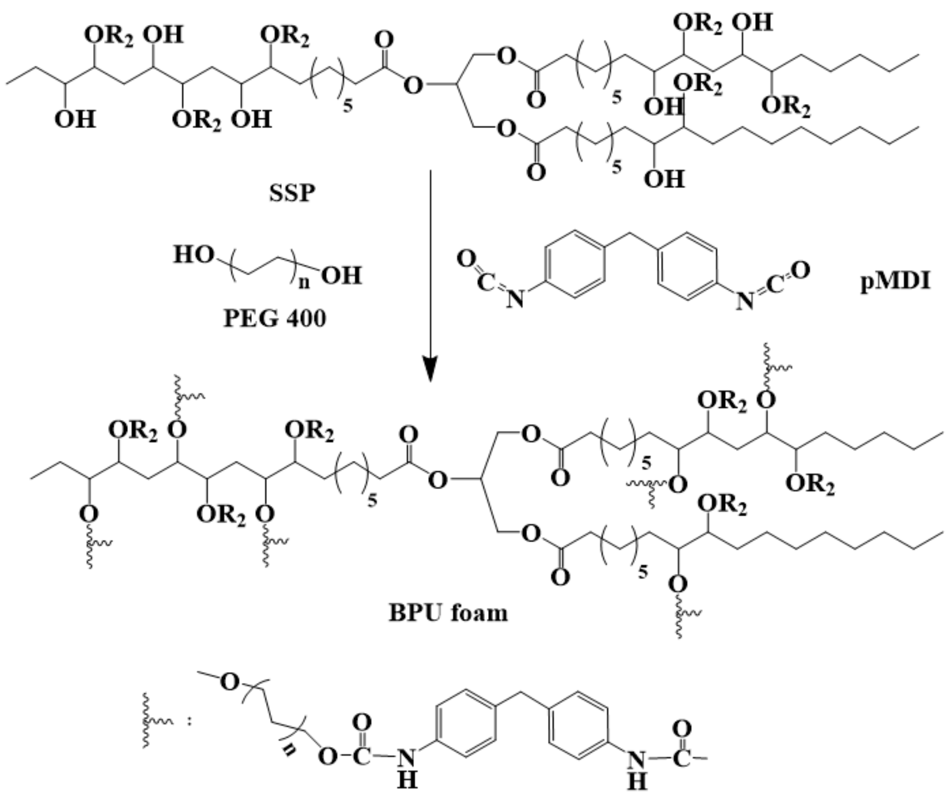

Scheme S1. Synthesis process of SSP-based BPUF

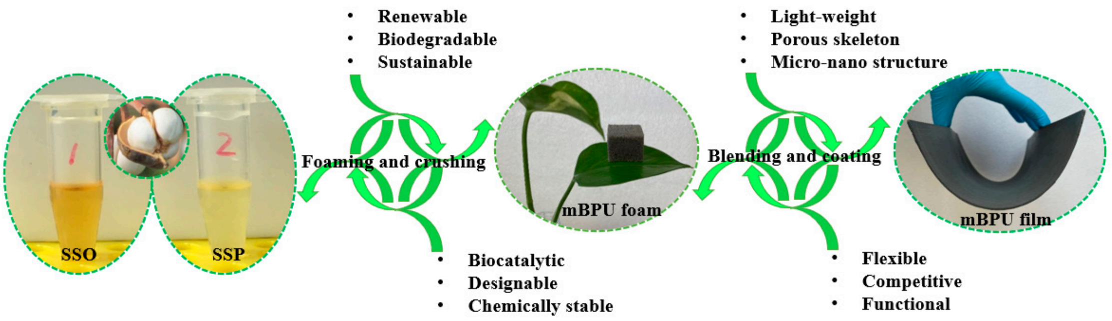

Scheme S2 Molding process of the mBPUF and mBPUF coating film composites

3. Electromagnetic performance mBPUF coating film

Figure S2 displays the test method and process of reflectivity loss in the mBPUF and mBPUF film. The vertical reflectance of the composite is calculated in the vector network analysis (VNA) system. The mBPUF with a 30% mass fractionin paraffin was sliced into a coaxial ring (Φ out : 7 mm, Φ in : 3.04 mm) at a thicknessof 2.0 mm for VNA measurement. The standard size of the base plate and mBPUF film is 180

mm×180 mm, and the average thickness of the composite films is 2.0 mm. Calibration is required before testing.

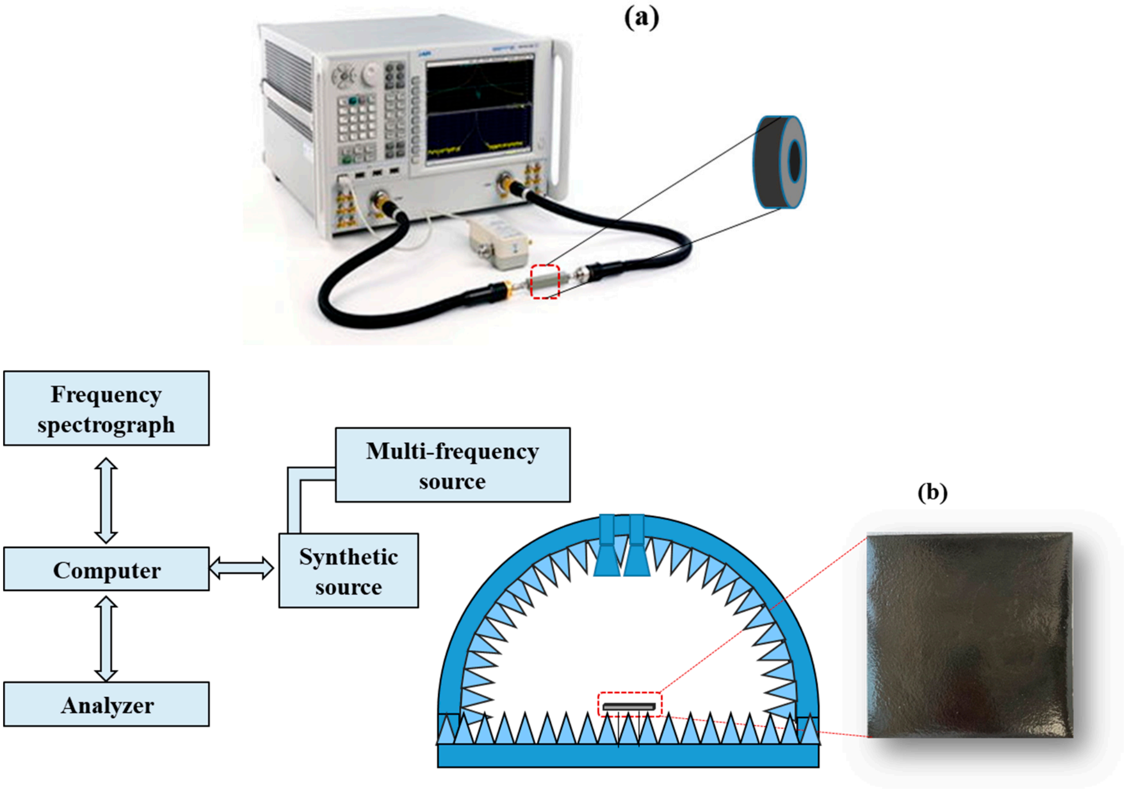

Figure S1. Test method of reflectivity loss in the(a)mBPUF and(b)mBPUF filmcomposite

4. Microstructure construction of $Fe_3O_4$and its mBPUF composites

The microstructure and dynamic blending model images of Fe_3O_4 and its mBPUF film are constructed using the molecular dynamic (MD) simulations to elucidate the structural effect on the characters of the mBPUF composite. From the results of the structure, Fe_3O_4 is octahedral granular microstructure with an orderly arrangement of thelattice structure. The system mixing of the mBPUF composite is performed in the Amorphous Cell module. Numerous interfaces exist between the Fe_3O_4 and the BPUF in the skeleton. The density distribution of the composite

displays a funnel form molding for the encasing of the Fe_3O_4 in the BPUF matrix. This unique structure may generate the eddy current effect under alternating the electromagnetic field.

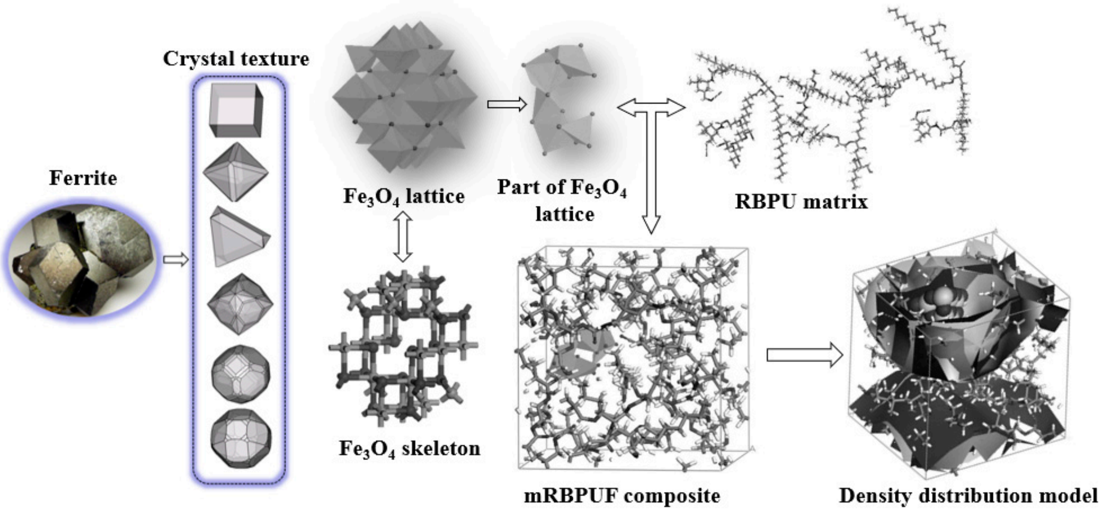

Figure S2. Microstructure and dynamic blending model images of Fe_3O_4 and its mBPUF composites
